# Detection of Terbufos (Organophosphate) Intoxication in Necrophagous Larvae Using Biospectroscopy (ATR-FTIR) with Multivariate Selection Algorithms

**DOI:** 10.1007/s13744-026-01430-6

**Published:** 2026-09-02

**Authors:** Jéssica Teixeira Jales, Taciano de Moura Barbosa, Hellyda Katharine Tomaz de Andrade Silva, Kassio Michell Gomes De Lima, Vanessa de Paula Soares, Renata Antonaci Gama

**Affiliations:** 1 Forensic Genetics Lab, Forensic Expert at the Scientific Police of Rio Grande Do Norte (PCI-RN), Natal, Rio Grande Do Norte Brazil; 2 Lab of Insect and Vectors, Dept of Microbiology and Parasitology, Federal Univ of Rio Grande Do Norte, Natal, Rio Grande Do Norte Brazil; 3 Dept of Biophysics and Pharmacology, Federal Univ of Rio Grande Do Norte, Natal, Rio Grande Do Norte Brazil; 4 Dept of Chemistry Natal, Institute of Chemistry, Federal Univ of Rio Grande Do Norte, Natal, Rio Grande Do Norte Brazil

**Keywords:** *Lucilia eximia*, Spectroscopy, Pesticide, Forensic entomology, FTIR

## Abstract

Entomotoxicology studies the effect and detection of different xenobiotics in scavenger insects, aiding in the correct estimation of the interval and cause of death. Terbufos (organophosphate) stands out in the forensic field for its association with intoxication cases. However, the methodologies for its detection remain laborious, expensive, and destructive for crime clues, with the need for simpler, accessible, and conservative tools for detection of this compound in the entomological evidence. Thus, this study aims to evaluate (i) Attenuated Total Reflection-Fourier Transform Infrared (ATR-FTIR) as a tool to differentiate larvae collected from negative control carcasses and those treated with terbufos and (ii) the overall sensitivity and specificity of the technique for identifying terbufos exposure in forensic contexts, discussing its applicability in real cases of organophosphate poisoning. For this, *Lucilia eximia* (Diptera: Calliphoridae) larvae (*n* = 120; 30/group), collected from rat carcasses previously treated with 5 (T1), 10 (T2), or 20 (T3) mg/kg of terbufos, as well as from untreated control carcasses (negative control—NC), were used for spectra collection and standardization of classification model. For data analysis, differentiation, and classification, unsupervised (PCA) and supervised (PCA-LDA, GA-LDA, and SPA-LDA) analysis algorithms were used. The PCA showed a tendency of differentiation between the analyzed groups. However, only with the supervised analysis algorithms, spectra from the negative control, T1, T2, and T3 groups were differentiated. The PCA-LDA and SPA-LDA algorithms specially differentiated the T3, T2, and NC groups, reaching an average 85% sensitivity and 90% specificity, while the GA-LDA showed 100% sensitivity and specificity, being the model of choice for analyzing this dataset. The main variables listed for sample differentiation were in the regions of protein phosphorylation, phosphate stretch, and protein content. Thus, this study showed that the ATR-FTIR coupled with multivariate analysis algorithms can efficiently differentiate larvae collected from carcasses non-intoxicated or intoxicated with different doses of terbufos, representing a viable, fast and efficient screening alternative for entomotoxicology.

## Introduction

Entomotoxicology studies the influence and detection of toxic substances in insects associated with decomposing carcasses or corpses (Introna et al. 2001), contributing to the prevention of errors in civil or criminal investigations involving toxicants. Its relevance is evident when considering that the accuracy of entomological evidence and data can be significantly altered in the presence of narcotics or pesticides (Catts and Goff 1992; Hou S et al. 2025). Toxic substances can alter the process of cadaveric decomposition, attraction, life cycle duration, and survival of carrion-associated insects (Abd El-Bar and Sawaby 2011; De Siqueira et al. 2016; Jales et al. 2020; 2021a, b). Additionally, the detection of chemicals within insects has contributed not only to the determination of the post-mortem interval (PMI), but also to their direct association with the cause of death (Gunatilake and Goff 1989; Goff and Lord 1994; Hou S et al. 2025; Beltrame et al. 2026).

Among pesticides, carbamates and organophosphates lead intoxication cases in Brazil and worldwide (EPA 2013; Silva et al. 2016; Eddleston 2019), with a particular emphasis on terbufos (organophosphate), an insecticide frequently associated with suicide cases in Brazil (Ferrari Junior and Caldas 2018; Magalhães and Caldas 2018). Terbufos (S-tert-butylthiomethyl O,O-diethyl phosphorodithioate), popularly known in Brazil as “chumbinho,” has had its use discontinued due to its high toxicity to humans and other animals, in addition to contaminating soil, water, and other environments (Bowman and Sans 1982). Once in the organism, terbufos is metabolized by cytochrome oxidase and glutathione S-transferase enzymes in mammals and insects (Espinoza-Navarro 2017). Organophosphates act through the irreversible phosphorylation of the acetylcholinesterase enzyme, causing acetylcholine accumulation in the synaptic clefts and the subsequent appearance of cholinergic overexpression symptoms (dizziness, tremors, cough, sweating) (EPA 2013).

Organophosphates such as terbufos cause alterations in the decomposition process of animal carcasses (Abd El-Bar and Sawaby 2011; De Siqueira et al. 2016; Jales et al. 2020), in the developmental cycle duration of colonizing insects (Jales et al. 2020), in larval and pupal mass (Carvalho et al. 2001), fecundity (Hunter et al. 1958; Bariola 1984), or adult longevity (Hamilton and Schal 1990). Furthermore, they induce involuntary body contractions and mobility changes in invertebrates (Haynes 1988; De França et al. 2017), representing an important investigative tool for forensic entomology (Catts and Goff 1992) and entomotoxicology (Introna et al. 2001) in intoxication cases. Therefore, detecting chemical compounds in insects associated with decomposing remains provides crucial evidence for determining the cause of death (Goff and Lord 1994). However, current analytical methods like gas chromatography–mass spectrometry (GC-MS) (WHO 2003) are costly, time-consuming, and destroy sample integrity.

In this context, simpler, faster, and more accessible tools such as spectroscopic techniques may represent viable alternatives for identifying terbufos traces in necrophagous fly larvae. According to Jales et al. (2021a, b), spectroscopic techniques have proven useful in various fields of entomology, although they remain under-explored in forensic entomology and entomotoxicology studies. Spectroscopic methodologies, such as biospectroscopy, utilize vibrational patterns resulting from the interaction of chemical and biological specimens with electromagnetic light (Fifield and Kealey 2000; Santos et al. 2017). One of the infrared spectrum regions used for determining biological components is the mid-infrared (MIR—4000–400 cm^−1^). MIR instruments emit an electromagnetic light beam at specific wavelengths, which interacts directly with the sample, generating vibrational and torsional chemical changes in the molecules such as stretching, bending, and twisting that are specific and inherent to each sample (Davis and Mauer 2010).

In the application of MIR for the analysis of biological components, two spectral regions stand out: the double-bond region and the biological fingerprint region. The former (2000 to 1500 cm^−1)^ is associated with C=C, C=N, and C=O stretching, while the biological fingerprint region (1800 to 900 cm^−1^) identifies molecular skeleton bending and vibrations. The biochemical information associated with this region provides a representation of the structure and function of cellular constituents based on vibrational bands (Chiriboga et al. 2000; Kelly et al. 2011; Martin et al. 2007), yielding specific information on components such as lipids, carbohydrates, proteins, DNA/RNA, and asymmetric and symmetric stretching vibrations of methylene and phosphate groups (Kelly et al. 2011; Martin et al. 2007).

The application of infrared techniques generates a massive amount of data to be analyzed and interpreted. To assist in pre-processing, sample classification, and dimensionality reduction, algorithms have been developed as key components of chemometrics (Hibbert 2016) and multivariate data analysis (Johnson and Naiker 2019). Principal component analysis (PCA) stands out as a tool for pattern detection and data dimensionality reduction (Abu-Mostafa 2012). Conversely, Genetic Algorithm (GA), Successive Projections Algorithm (SPA), and Linear Discriminant Analysis (LDA) are highlighted as supervised analysis tools (Soares et al. 2012; McCall 2005) and have already been successfully applied to insect identification and forensic drug detection (Ali et al. 2011; Oliveira et al. 2014; Baia et al. 2016; Barbosa et al. 2018).

Given the need for simple and fast tools to detect chemical substances in insects, we evaluated ATR-FTIR to discriminate *Lucilia eximia* (Diptera: Calliphoridae) larvae exposed to terbufos-intoxicated carcasses from non-exposed larvae (negative controls). Specifically, we sought to (i) investigate the capacity of ATR-FTIR, combined with classification algorithms, to differentiate larvae collected from non-intoxicated carcasses from those exposed to terbufos and (ii) assess the overall sensitivity and specificity of the technique for identifying terbufos exposure in forensic contexts. We hypothesized that ATR-FTIR spectral fingerprints allow for the accurate detection of terbufos intoxication in larvae, even at low exposure levels.

## Material and Methods

### Experimental Design and Larval Collection

Experiments were conducted using larvae of *Lucilia eximia* (Wiedemann) (Diptera: Calliphoridae), collected from non-intoxicated and terbufos-intoxicated rat carcasses. *L. eximia* was selected once it is one of the dominant species in the colonization process of rat carcasses intoxicated with terbufos in an Atlantic Forest fragment in Rio Grande do Norte, Brazil (05°50′33.3″S 35°12′05.6″W) (Jales et al. 2021a, b).

The terbufos (organophosphate, unknown purity) used in this study was obtained and made available by the Laboratory of Forensic Analysis and Research of the Rio Grande do Norte Technical-Scientific Police Institute (ITEP-RN) (letter No. 160/2019) and its preparation and solubilization followed Jales et al. (2020).

To collect the larval specimens, female rats (*n* = 3 carcasses per group, *n* = 4 groups; CEUA approval no. 018/2019) were divided into non-intoxicated (negative control, NC) and terbufos-intoxicated (5 mg/kg (T1), 10 mg/kg (T2), and 20 mg/kg (T3)) groups, following the tolerance range established by the United States Environmental Protection Agency (EPA 2013). The rats were intoxicated by gavage (200 µL final volume) and 30 min following administration were euthanized by cervical dislocation. This period was required for the terbufos to be metabolized, reach the bloodstream of the intoxicated animals (EPA 2013) and equally reach their tissues. No other body lesion was caused to the animals after terbufos administration once tissue lesions could compromise the decomposition process. In addition to metabolization time, signs and symptoms of organophosphate intoxication (i.e., bristling fur, tremors, increased salivation, disorientation) were identified in all intoxicated rats. The animals were then exposed in suspended traps as proposed by Jales et al. (2020) and adapted from Carmo et al. (2017). The suspended traps used in this study allowed the collection of immatures in dispersion, that is, third instar larvae that have reached maturity and dispersed from the carcasses.

The traps containing the dispersing immatures were transported to the Laboratory of Insects and Vectors at the Federal University of Rio Grande do Norte. The larvae were euthanized by freezing and subsequently selected and identified using the identification keys provided by Oliveira-Costa (2013). Following identification, 30 *L. eximia* larvae from different carcasses of each group (NC, T1, T2, and T3) were separated into containers and designated for spectral acquisition. The larvae were maintained at −20°C without preservatives to avoid chemical interactions.

### Spectral Acquisition

Larvae at room temperature were individually placed, using straight-tipped forceps, onto the crystal of a Bruker FTIR spectrophotometer (Model: VERTEX 70) equipped with an attenuated total reflectance (ATR) accessory at the Laboratory of Multifunctional Materials and Numerical Experimentation of the Federal University of Rio Grande do Norte.

**Fig. 1:**
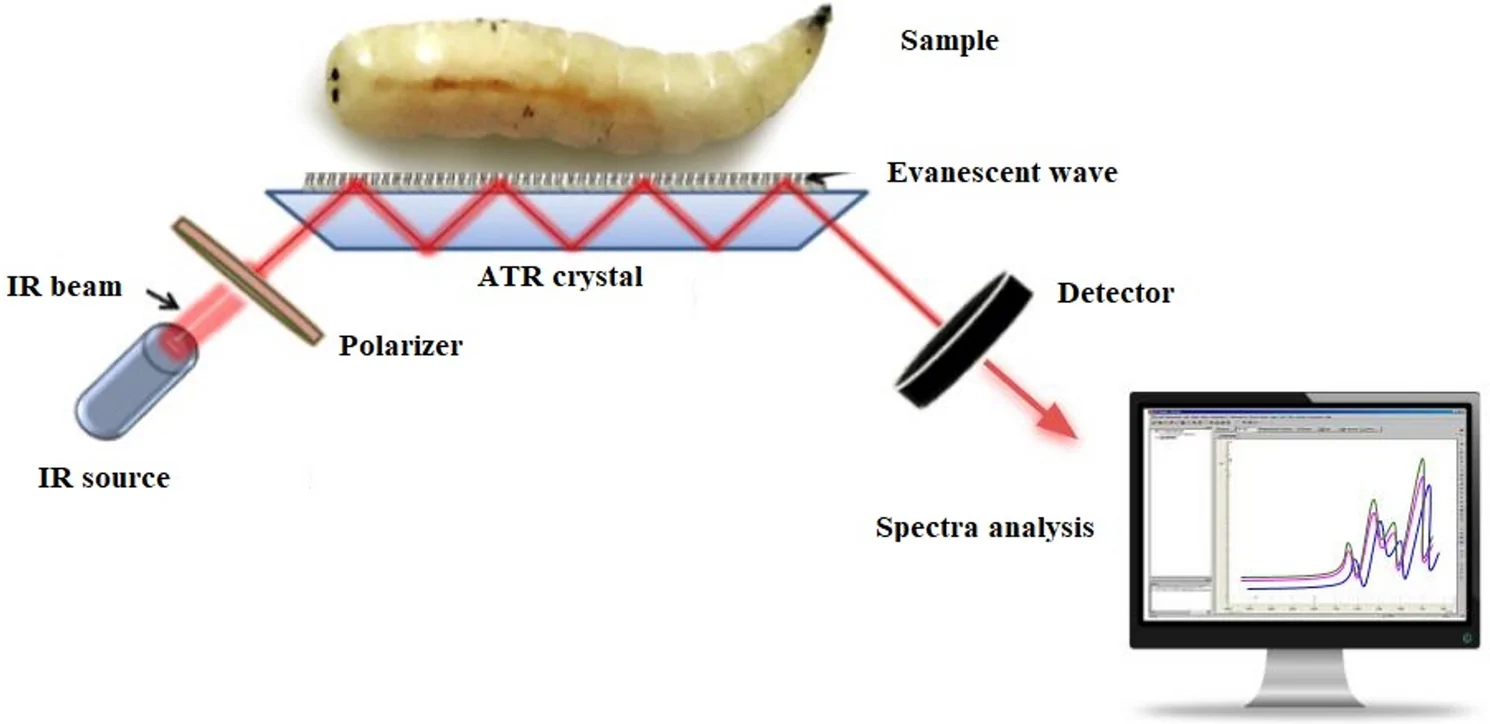
Schematic showing the positioning and collection of sample spectra, using a larva as an example. The proportion between images is for illustrative purposes only

Spectra were collected from the anterior, middle, and posterior regions of the larvae, as illustrated in Fig. 1, to characterize analytical variability and improve the robustness of the spectral dataset. Each spectrum was acquired independently and reflects local chemical information from the measured sample region. Spectra were obtained in the range of 400 to 4000 cm^−1^ with a resolution of 4 cm^−1^, using 16 scans with a total measurement time of 2 s per spectrum, totaling 360 larval spectra (30 samples × 4 groups × 3 different larval regions). Before the spectral acquisition of each specimen, the ATR crystal was cleaned with 70% (v/v) alcohol and a new background spectrum was obtained to ensure the absence of residual interferences. The measurements were carried out in a climate-controlled laboratory, under stable temperature (approximately 22 °C) and relative humidity conditions. Room air was used as the background spectrum, as the specimens remained in contact with the room air during spectral acquisition. It is also worth noting that the inclusion of multiple larvae and spectra allowed the model to capture both biological and analytical variation that would be expected in practical forensic entomology applications, where individual larval specimens rather than carcasses constitute the material available for analysis.

### Pre-processing and Multivariate Analysis of Spectral Data

Raw spectra were pre-processed to remove external interferents. Each spectrum was truncated to the 900 to 1800 cm^−1^ range, corresponding to the biological fingerprint region, followed by Automatic Weighted Least Squares baseline correction and smoothing using the Savitzky-Golay filter (15 points filter width, 2nd order polynomial fitting), performed with MATLAB R2014b software (MathWorks, Natick, USA). No spectral normalization or scaling was needed. After pre-processing, the spectra underwent unsupervised (exploratory) and supervised data analysis using mathematical models. It is important to mention that spectra were treated as the analytical units for dataset partitioning and model construction.

The exploratory analysis of the dataset was performed using PCA to identify patterns and clustering trends (e.g., larvae from the NC and terbufos-intoxicated groups) related to chemical similarities or dissimilarities among the samples (Morais et al. 2020). This technique reduces dimensionality and enables better data visualization by separating data solely based on spectral variability (Kelly et al. 2011). PCA corresponds to a linear transformation of the dataset wavelengths through loading matrices, also known as principal components (PCs) or loading vectors. To visualize the data in 2D Fisher score plots, the loading vectors are combined in orthogonal pairs (90° to each other), representing individual samples.

The PCA was based on the first four principal components, which covered 99.8% of the cumulative explained variance. It is worth mentioning that the number of PCs was chosen based on the cumulative explained variance criterion, retaining the minimum number of components that captured the maximum amount of meaningful spectral information.

### Supervised Chemometric Analysis

In supervised methods, algorithms are trained to recognize discriminant patterns (Beebe 1998). The main advantage of this approach is that once the model is consistently built using training, validation, and testing phases, it can be saved and reused multiple times for the classification of unknown samples (Abul-Mostafa 2012). LDA is a supervised linear transformation method that projects the variables (wavenumbers, in the case of FTIR) that best delimit and explain the mathematical model. Similar to PCA, the LDA vectors are also orthogonal (at 90° to each other) and maximize the variance between classes (Hastie et al. 2010).

Sample variables or factors represent the linear combination between wavelength and sample absorbance (Martin et al. 2007). For the application of LDA, variables must be previously selected (Silva et al. 2013). In this study, an algorithm for data dimensionality reduction (PCA) was used, along with two others for variable selection: SPA and GA combined with the linear discriminant function (LDA).

In the PCA-LDA, SPA-LDA, and GA-LDA models, each group (control, T1, T2, and T3) was treated separately. Samples were divided into training (70% of spectra), validation (15% of spectra), and testing (15% of spectra) sets for the qualitative evaluation of the model’s sample discrimination, applying the classic Kennard-Stone (KS) uniform sampling algorithm (Kennard and Stone 1969). The optimal number of variables for SPA-LDA and GA-LDA was determined by the minimum cost of the G function (2), calculated for a given validation sample set using the equation:

1 $$G=\frac{1}{{N}_{v}}{\sum}_{n=1}^{{N}_{v}}{g}_{n}$$

where *g*_*n*_ (3) is defined as

2 $${g}_{n=\frac{{r}^{2}\left({x}_{n},{m}_{I\left(n\right)}\right)}{{min}_{I\left(m\right)\ne l\left(n\right)}{x}^{2}\left({x}_{n},{m}_{I\left(n\right)}\right)}}$$

where *I*(*n*) is the true class index for the *n*^th^ validation object *n*.

In the GA-LDA model, mutation and reproduction probabilities were kept constant at 10% and 80%, respectively, with an initial population of 60 individuals and 30 generations. The resulting solution after three runs of the GA-LDA was chosen. Additionally, the scores, loadings, and discriminant function (DF) values from the LDA were derived from the biological fingerprint of the larvae, whether intoxicated with terbufos or not. The first DF was used to visualize sample alterations in (1D) score plots to represent the primary chemical changes.

### Qualitative Analysis of Supervised Models

For the qualitative analysis of the supervised models, the figures of merit were calculated according to Baia et al. (2016), which included sensitivity (the confidence that a positive result is obtained for a positive sample); specificity (the confidence that a negative result is obtained for a negative sample); positive predictive value (PPV; the proportion of positive examples correctly assigned, with values ranging from 0 to 1); negative predictive value (NPV; the proportion of negative examples correctly assigned, with values ranging from 0 to 1); the *Youden* index (YOU; which evaluates the classifier's ability to avoid failures); and the positive likelihood ratios (LR+; representing the ratio between the probability of predicting an example as positive when it is truly positive, and the probability of predicting it as positive when it is actually non-positive) and negative likelihood ratios (LR−; representing the ratio between the probability of predicting an example as negative when it is truly positive, and the probability of predicting it as negative when it is actually negative).

## Results

**Fig. 2:**
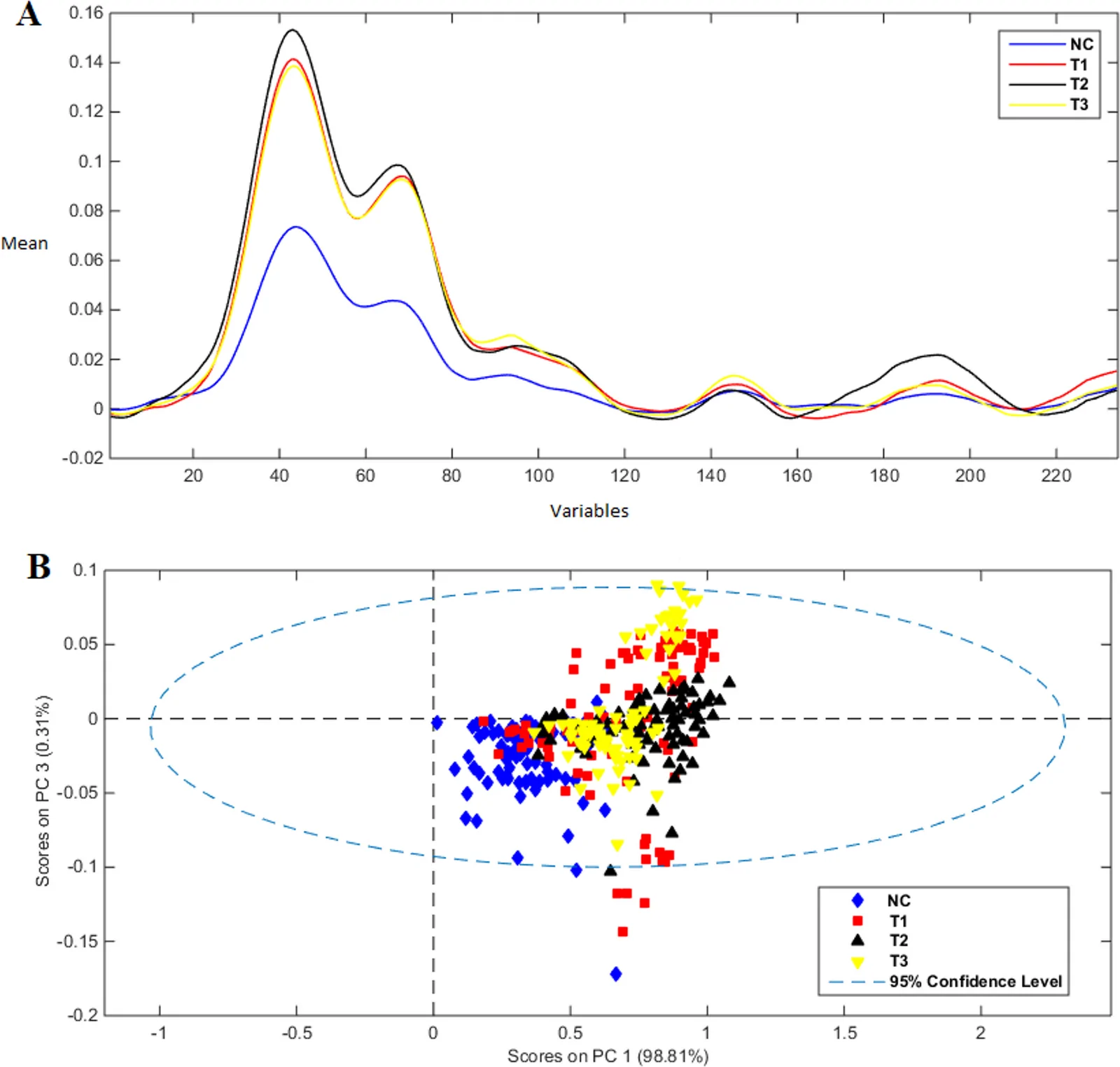
Graphical representation of spectral averages and PCA. **A** Average spectra of the analyzed groups and **B** 2D Fisher score plot resulting from the overlap of components PC1 and PC3 for PCA analysis. NC, negative control (blue); T1, treatment with 5 mg/kg of terbufos (red); T2, treatment with 10 mg/kg of terbufos (black); T3, treatment with 20 mg/kg of terbufos (yellow)

A total of 360 spectra were collected from larvae collected from non-intoxicated and intoxicated carcasses. The pre-processed average spectra for each class (NC, blue line; T1, red line; T2, black line; T3, yellow line) within the biological fingerprint region are shown in Fig. 2A. However, in the regions of variables 40, 70, 100, 150, and 170, the spectra were shifted upwards, indicating higher absorbance in these specific regions.

Principal component analysis (PCA) selected four principal components as sufficient to explain the data variability. The first component (PC1) accounted for approximately 98.8% of the total variance, while the second, third, and fourth components explained 0.56%, 0.31%, and 0.13%, respectively. Figure 2B displays the 2D Fisher score plot for the PCA, where each point represents an individual spectral sample. Based on the score graph, the PCA showed a trend in the separation between the negative control group and the intoxicated groups (T1, T2, and T3), regardless of the dose. However, a satisfactory separation was not observed, as there was overlap between some samples from the control group and the intoxicated groups.

**Fig. 3:**
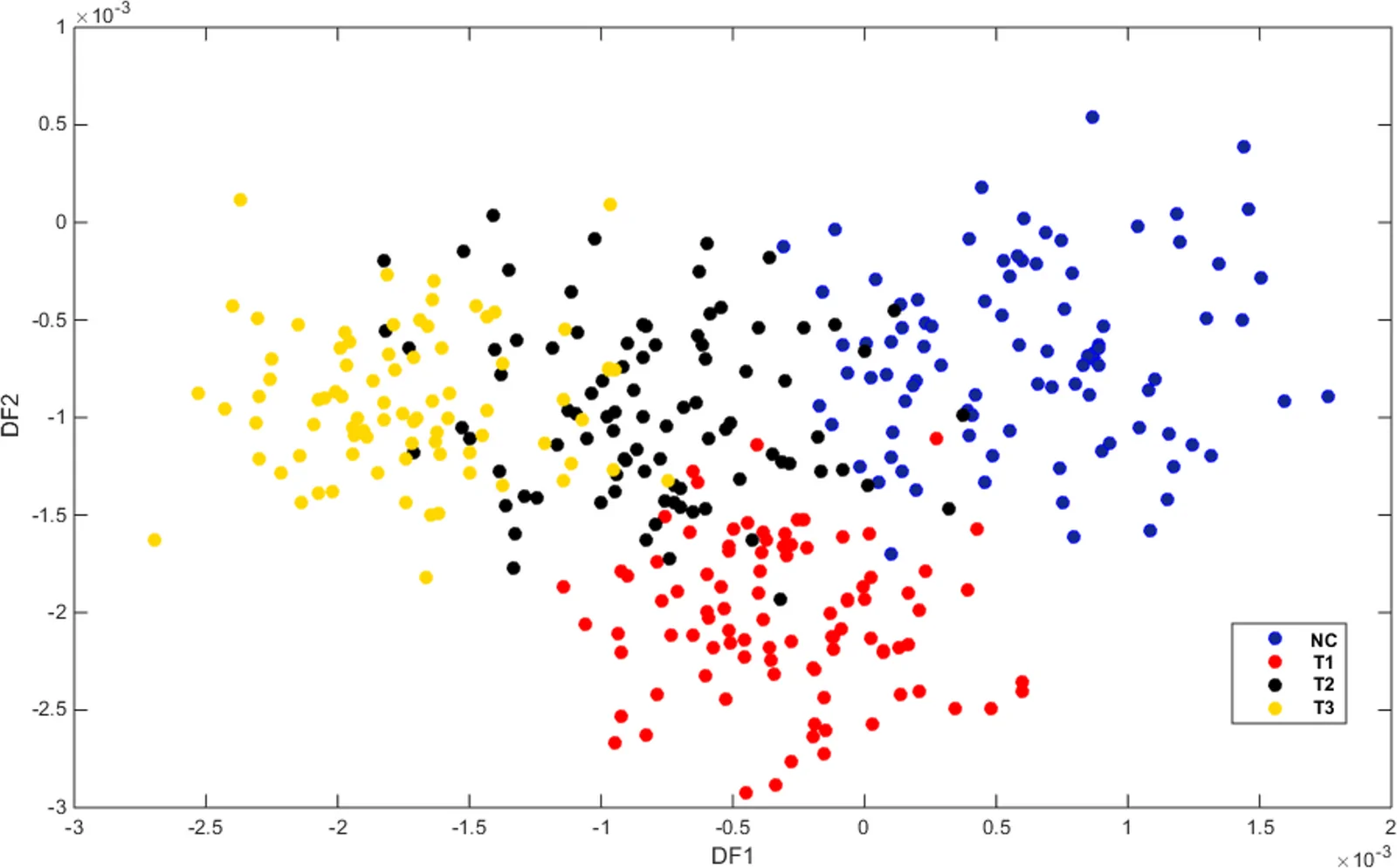
Discriminant functions calculated for the differentiation between negative control (NC—blue), T1 (5 mg/kg—red), T2 (10 mg/kg—black), and T3 (20 mg/kg—yellow) using PCA-LDA

The 2D Fisher score plot for PCA-LDA (Fig. 3) displays the spectral points derived from the samples. The PCA-LDA model was built using five principal components (98% cumulative variance) considering the classification performance and exhibited a segregation trend between classes, especially between the negative control and the terbufos-intoxicated groups. Although PC5 contributed only marginally to the total explained variance, its inclusion improved the discriminatory performance of the supervised classification model. Furthermore, the overlap of the DF1 and DF2 vectors demonstrates high intra-class sample homogeneity, with the negative control being visually separated from the other terbufos-intoxicated groups, regardless of the dose.

**Fig. 4:**
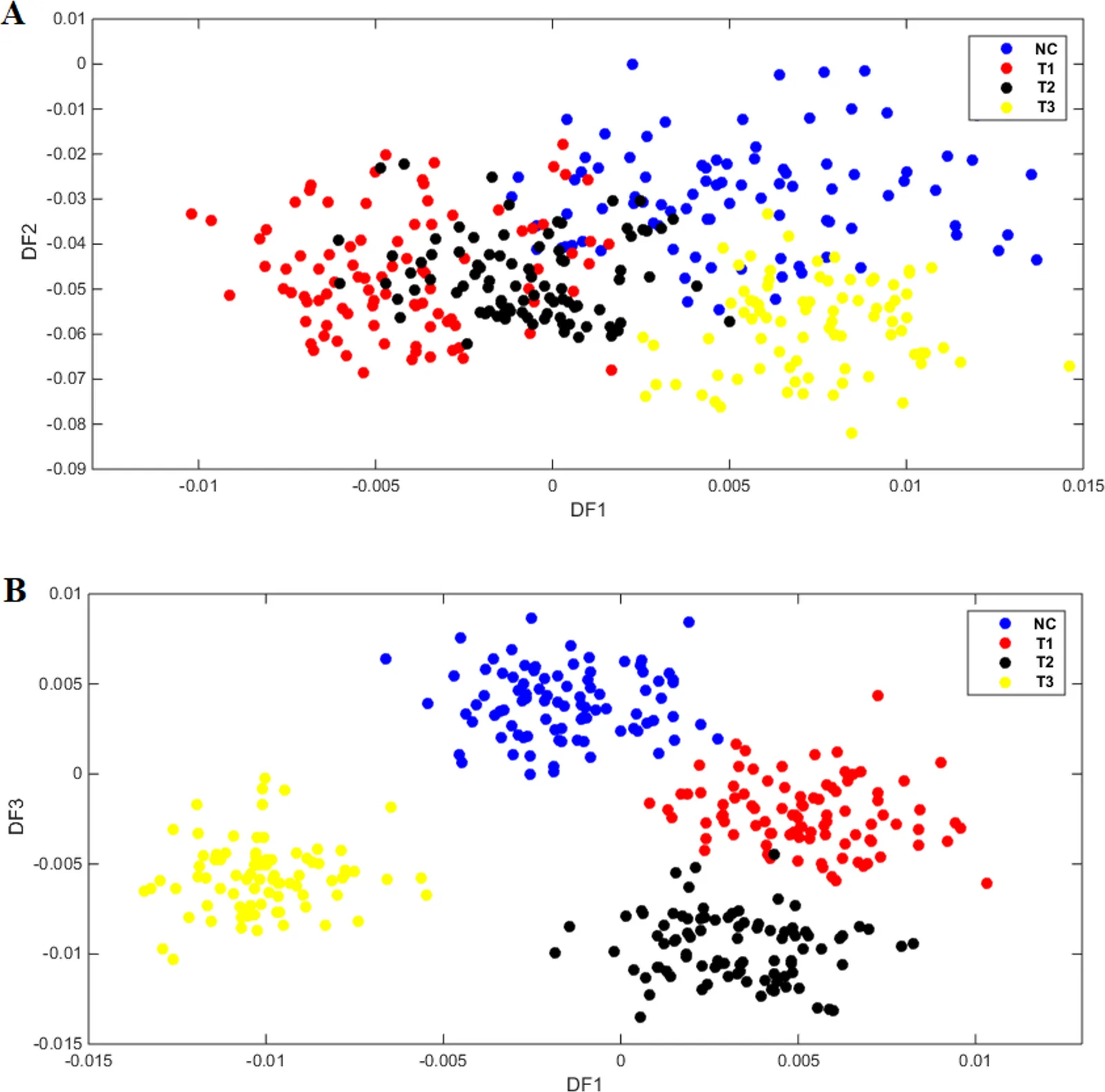
Discriminant functions calculated for the differentiation between negative control (blue), T1 (5 mg/kg—red), T2 (10 mg/kg—black), and T3 (20 mg/kg—yellow) using **A** SPA-LDA and **B** GA-LDA

SPA-LDA and GA-LDA were applied to the dataset to obtain the optimal number of variables through the minimum cost in the G function scores. The discriminant function analysis plots show similar efficiency between PCA-LDA (Fig. 3) and SPA-LDA for sample separation (Fig. 4A). In this model, the groups maintained spectral homogeneity, particularly the terbufos-intoxicated groups. The GA-LDA showed optimal efficiency for sample separation, as observed in Fig. 4B. In this model, all groups were discriminated, regardless of the dose or the presence of terbufos.

**Fig. 5:**
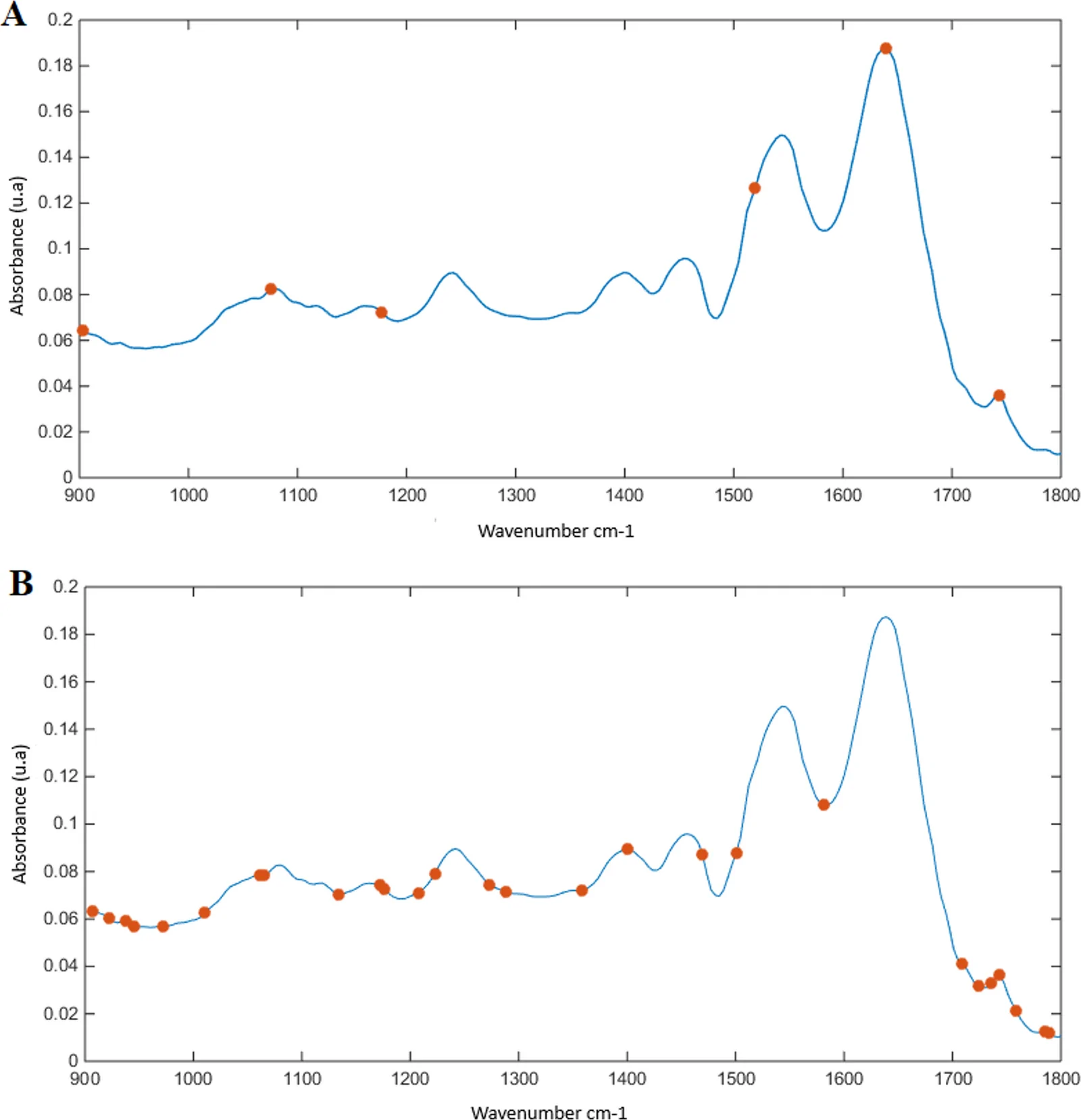
Variables selected for the construction of the **A** SPA-LDA and **B** GA-LDA models

SPA-LDA resulted in the selection of six variables (Fig. 5A) used to calculate the Fisher scores for the four sample classes. Analysis of the selected variables showed that the primary biochemical alterations for class discrimination in the SPA-LDA model were proteins and nucleic acids, specifically the protein phosphorylation region (902 cm^−1^), symmetric and asymmetric phosphate stretching vibrations (1076 cm^−1^), Amide I (1520 cm^−1^), Amide II (1639 cm^−1^), and lipids (1743 cm^−1^). However, there was no significant improvement compared to the PCA-LDA model, and both exhibited similar classificatory power.

The GA-LDA model selected 26 variables for calculating the Fisher scores (Fig. 5B), resulting in an increase in within-class homogeneity and between-class discrimination (Fig. 4B). Analysis of the selected variables revealed that, similar to the SPA-LDA model, regions related to proteins and nucleic acids—protein phosphorylation (~970 cm^−1^), symmetric and asymmetric stretching of phosphate groups (~1080 cm^−1^, ~ 1225 cm^−1^), Amides II (1580 cm^−1^) and III (1273 cm^−1^), and lipids (~1750 cm^−1^) were crucial for sample discrimination (Fig. 5B).

Qualitative analysis of the models showed an average sensitivity of 81.6% for correct sample classification when PCA-LDA was used, exhibiting higher sensitivity for differentiating between groups T3, NC, and T2, respectively, and an average specificity greater than 90% (Table 1). SPA-LDA yielded an average sensitivity of approximately 85% and specificity greater than 90%, similar to the results achieved by PCA-LDA. In contrast, GA-LDA achieved 100% sensitivity and specificity for differentiating between the studied groups, representing the best model to be applied to this dataset, regardless of the presence or dose of terbufos.

**Table 1:** Figures of merit for the PCA-LDA, SPA-LDA, and GA-LDA classification models used to categorize the negative control (NC), treatment 1 (T1—5 mg/kg terbufos), treatment 2 (T2—10 mg/kg terbufos), and treatment 3 (T3—20 mg/kg terbufos) groups. *PPV*, positive predictive value; *NPV*, negative predictive value; *YOU, *Youden index; *LR+*, positive likelihood ratio; *LR−*, negative likelihood ratio

Algorithm	Figures of merit	CN	T1	T2	T3
**PCA-LDA**	Sensitivity	80	66,6	80	100
	Specificity	95,3	97,6	90,6	90,6
	PPV	0,85	0,9	0,75	0,78
	NPV	0,93	0,89	0,92	1
	YOU	75,3	64,2	70,6	90,6
	LR+	17,02	27,75	8,51	10,63
	LR-	0,058	0,036	0,117	0,094
** SPA-LDA**	Sensitivity	73,3	80	86,6	100
	Specificity	95,3	95,3	93	95,3
	PPV	0,84	0,85	0,81	0,88
	NPV	0,91	0,93	0,95	1
	YOU	68,6	75,3	79,6	95,3
	LR+	15,59	17,02	12,37	21,27
	LR-	0,064	0,058	0,08	0,047
**GA-LDA**	Sensitivity	100	100	100	100
	Specificity	100	100	100	100
	PPV	1	1	1	1
	NPV	1	1	1	1
	YOU	100	100	100	100
	LR+	-	-	-	-
	LR-	-	-	-	-

## Discussion

Spectroscopic techniques have been gaining ground as alternative tools in forensic entomology (Jales et al. 2021a, b), particularly in the detection and differentiation of drugs for formulation adulteration analysis (Ryan et al. 1991), smuggling (Scafi and Pasquini 2001; He et al. 2020), or detection in human tissues and scavenger insects (Ali et al. 2011; Oliveira et al. 2014; de Lima et al. 2014a, b; Baia et al. 2016). In this study, ATR-FTIR coupled with multivariate data analysis techniques was used as a screening tool to discriminate larvae collected from non-intoxicated and intoxicated rat carcasses with terbufos, an illegally traded organophosphate, highly toxic, and frequently used in suicide cases in Brazil (Ferrari Junior and Caldas 2018; Magalhães and Caldas 2018). The supervised analysis algorithms, especially the GA-LDA, showed 100% sensitivity and specificity in discriminating non-intoxicated larvae from terbufos-exposed groups, even at the lowest concentration tested (5 mg/kg), thus representing a rapid, low-cost, and non-destructive screening tool capable of identifying biochemical alterations associated with intoxication patterns. These results corroborate our hypothesis that the technique combined with multivariate analysis algorithms, particularly GA-LDA, possesses the necessary sensitivity and specificity to identify traces of terbufos mono-intoxication, even at low concentration (5 mg/kg).

Among the chemometric approaches used, PCA was selected as an important tool to reduce the dimensionality and complexity of spectral data into variables capable of explaining data variability (Kelly et al. 2011). PCA was utilized by Baia et al. (2016) as an alternative for detecting flunitrazepam in *C. megacephala* larvae originating from carcasses intoxicated with the compound. However, as a standalone model, PCA was not efficient for spectral differentiation. Similarly, PCA alone did not clearly differentiate the intoxicated groups, although it did show a separation trend between intoxicated and non-intoxicated specimens (Fig. 2B). The low efficiency of the unsupervised method in distinguishing the intoxicated groups from the negative control may result from factors such as the concentration of terbufos within the larvae, a parameter directly influenced by carcass decomposition time and bioavailability of the compound in the rodent tissues, as well as its accumulation capacity in larval tissues. However, this hypothesis still remains to be tested.

The use of spectroscopic tools represents an important technical advancement for entomotoxicology (Jales et al. 2021a, b), a field of forensic entomology that establishes a consistent association between chemical compounds and the death of an individual through the analysis of necrophagous insects (Introna et al. 2001). The detection of these substances typically relies on a suite of chemical and biochemical techniques that require significant laboratory and technical support (Stoytcheva and Zlatev 2011). However, streamlining this process through the standardization of chemometric tools, such as applying classification algorithms to infrared spectral datasets, reduces equipment and consumable costs, minimizes the need for specialized personnel, and preserves the integrity of samples collected at crime scenes. These samples are crucial evidence for forensic counter-analysis.

In this study, coupling PCA with LDA to improve variable selection increased the model efficiency, resulting in more homogeneous classes and facilitating the visualization of spectral differentiation. Although PCA-LDA was not the model of choice for differentiating between non-intoxicated and flunitrazepam-intoxicated groups in previous research (Baia et al. 2016), it improved the classification of terbufos-intoxicated and negative control groups in the present work (Fig. 3). After dataset training and validation, PCA-LDA achieved an average sensitivity of 81% and an average specificity of 90% for correct group classification.

Biological samples exhibit high chemical complexity, and a single spectrum may present hundreds of variables to be analyzed and compared within or between classes. Consequently, more robust mathematical models must be applied to biological datasets to enhance the efficiency of variable selection and analysis. SPA coupled with LDA represents one of the mathematical tools frequently used in the selection and analysis of biological samples, such as in the differentiation of fly species (Barbosa et al. 2018), bacteria (Davis and Mauer 2010), fungi (Taha et al. 2013), and viruses (Lima et al. 2014a, b; Santos et al. 2017), as well as in the detection and differentiation of drugs in larval tissues (Oliveira et al. 2014; de Lima et al. 2014a, b; Baia et al. 2016). In this study, SPA-LDA demonstrated sample homogeneity and classificatory power similar to PCA-LDA, yielding an average sensitivity of 85% and an average specificity exceeding 90% for the classification of the different sample groups.

In contrast, the Genetic Algorithm (GA) significantly improved the homogeneity and classificatory power of the LDA model, achieving 100% sensitivity and specificity for the correct discrimination among sample groups. In the Fisher score plots (Fig. 4B), the formation of the four analyzed sample groups (NC; T1, T2, or T3) is clearly observable. Thus, GA-LDA was efficient in sample classification and represents the model of choice for analyzing the spectral data from larvae collected from terbufos-intoxicated rat carcasses. In models proposed by Oliveira et al. (2014), de Lima et al. (2014a, b), and Baia et al. (2016), GA-LDA also stood out among the multivariate analysis models utilized. GA is an algorithm with high optimization power for variable selection, originally inspired by natural selection (McCall 2005). In its mathematical process, the GA randomly selects and recombines variables based on a fitness process to produce a successor population (McCall 2005). As a result of this successive recombination process, the GA develops an optimal solution for a given complex problem, such as the differentiation of terbufos-intoxicated groups.

Among the variables selected by the SPA-LDA and GA-LDA models, the protein phosphorylation regions (900–970 cm^−1^), symmetric and asymmetric phosphate stretching vibrations (1070–1080 cm^−1^), proteins (Amide I (1520 cm^−1^) and Amide II (1580–1630 cm^−1^)), and lipids (1743–1750 cm^−1^) were highlighted for differentiating between the negative control group and the terbufos-intoxicated groups (Fig. 5). Terbufos acts through the irreversible phosphorylation of the acetylcholinesterase enzyme (EPA 2013), which breaks down acetylcholine. During terbufos intoxication, acetylcholinesterase receives a phosphate group that renders it inactive, leading to the accumulation of acetylcholine. This neurotransmitter remains active in the synaptic cleft, generating signs of cholinergic overstimulation characteristic of organophosphate poisoning—such as muscle spasms, sweating, dizziness, and nausea (EPA 2013). The variables related to protein phosphorylation and symmetric/asymmetric phosphate vibrations, as well as the high absorbances corresponding to protein groups (Amides II and III) selected by the SPA-LDA and GA-LDA models, can likely reflect general tissue responses and biochemical alterations associated with organophosphate exposure rather than direct quantification of the xenobiotic itself.

The high sensitivity and specificity associated with multivariate analysis algorithms experimentally demonstrate that ATR-FTIR can aid criminal investigations, as shown in the literature (Oliveira et al. 2016; de Lima et al. 2014a, b; Baia et al. 2016) and discussed by Jales et al. (2021a, b). In homicide cases with suspected intoxication, where entomological samples are the only available evidence for analysis, spectroscopy can reliably and rapidly determine the presence or absence of chemical substances. It is important to note that spectroscopic tools must be standardized and adapted to investigative routines regarding the different insect species and chemical compounds frequently encountered at crime scenes, especially in cases with multiple xenobiotics intoxication. This study is a preliminary proof-of-concept investigation under controlled experimental conditions, rather than as a validated forensic application, and it sheds light on how biospectroscopy can contribute to entomotoxicology and forensic entomology, empirically demonstrating that this is a promising union for intoxication with one xenobiotic, as suggested by Jales et al. (2021a, b).

The data presented in this study reinforce spectroscopy as a viable, efficient, and non-destructive alternative for identifying compounds commonly found in suicide cases in Brazil (Ferrari Junior and Caldas 2018; Magalhães and Caldas 2018). Jales et al. (2021a, b) discussed that spectroscopy was efficient in determining the age of insects colonizing carcasses, aiding in the estimation of the post-mortem interval (Pickering et al. 2015); in identifying necrophagous species (Barbosa et al. 2018); and in determining the cause of death by detecting substances ingested prior to death in blowfly larvae (Oliveira et al. 2014; de Lima et al. 2014a, b; Baia et al. 2016). This work shows the application of mid-infrared spectroscopy in the differentiation of *L. eximia* larvae, a dominant species in carcass colonization intoxicated with terbufos (Jales et al. 2020). Furthermore, this study demonstrates the efficiency of mathematical models, especially GA-LDA, in spectra differentiation, proposing it as the model of choice for evaluating this dataset. The identification of spectral markers related to proteins and phosphate groups provided a chemical basis for this discrimination, likely reflecting the biochemical impact of organophosphate intoxication.

## Data Availability

The raw datasets generated and analyzed during the current study are available from the corresponding author upon reasonable request, due to the lack of a dedicated institutional data repository at the authors' university.
